# Systematic Review and Meta‐Analysis of Acupuncture Treatment for Diabetic Cognitive Impairment: Focus on Animal Models

**DOI:** 10.1002/brb3.70783

**Published:** 2025-09-01

**Authors:** Xinyi Jiao, Yue Hu, Guoqing Tian

**Affiliations:** ^1^ Department of Traditional Chinese Medicine Peking Union Medical College Hospital, Chinese Academy of Medical Sciences and Peking Union Medical College Beijing China; ^2^ Department of Neurosurgery Peking Union Medical College Hospital, Chinese Academy of Medical Sciences and Peking Union Medical College Beijing China

**Keywords:** acupuncture, animal, diabetic cognitive impairment, meta‐analysis

## Abstract

**Background:**

Cognitive impairment is a frequent complication of diabetes, yet effective treatments remain elusive. In animal models, acupuncture has shown potential in improving cognitive deficits related to diabetes, but a comprehensive evaluation of its efficacy is lacking.

**Materials and Methods:**

We systematically searched seven databases (PubMed, Web of Science, Embase, OVID, SinoMed, CNKI, and Wanfang) from their inception through January 1, 2025. Studies meeting inclusion criteria underwent quality assessment using the SYRCLE Risk of Bias tool. Data analysis was conducted with Stata 14.0.

**Results:**

Thirteen studies comprising 294 animals were included. Acupuncture significantly reduced blood glucose in diabetic models [SMD = −2.44, 95% CI (−3.33, −1.55); *I* ^2^ = 88.9%, *p* < 0.000], shortened water maze escape latency [SMD = −2.35, 95% CI (−2.86, −1.84); *I* ^2^ = 60.0%, *p* = 0.003], and increased target platform crossings [SMD = 1.49, 95% CI (1.10, 1.88); *I* ^2^ = 51.9%, *p* < 0.001].

**Conclusion:**

Acupuncture can improve cognitive impairment in diabetic animal models and lower their blood glucose levels.

## 1 Introduction

Diabetes mellitus (DM) has emerged as a critical global health challenge, with its well‐established association with cognitive deterioration and dementia attracting substantial research attention (Srikanth et al. 2020). Population‐based studies have quantitatively demonstrated that DM independently increases the risk of both dementia and mild cognitive impairment by approximately 1.5‐ to 2‐fold (Kim et al. 2015; Biessels et al. 2006). The intricate mechanisms underlying cognitive decline in DM encompass chronic hyperglycemia, insulin resistance, vascular dysfunction, and inflammation, culminating in neuronal damage and cognitive impairment (Biessels and Despa 2018). Current therapeutic strategies primarily revolve around glycemic control, lifestyle modifications, and the management of associated vascular risk factors, yet their efficacy remains limited.

As a traditional Chinese medical intervention, acupuncture has gained recognition in evidence‐based medicine for its multimodal therapeutic effects (Chon and Lee 2013). Its therapeutic efficacy stems from the stimulation of specific acupoints to regulate the body's physiological processes and restore balance. Clinical studies have demonstrated promising results regarding the use of acupuncture in improving glycemic control and insulin sensitivity with DM (Chen et al. 2019). Furthermore, research has shown that acupuncture can enhance cognitive and memory function through mechanisms such as modulation of synaptic proteins, neuroinflammation, and increased hippocampal neuron density (X. Li et al. 2014; J. Li et al. 2024; X. Guo and Ma 2019; Su et al. 2020; Suh et al. 2019; G. Li et al. 2012). Currently, there have been many preclinical studies on acupuncture for the treatment of diabetic cognitive impairment (DCI), but their quality varies, and no meta‐analysis has been conducted to provide decision support for clinical research (American Diabetes Association 2021).

Meta‐analyses provide robust evidence regarding the efficacy and safety of acupuncture interventions for DM and associated cognitive impairments. Importantly, preclinical meta‐analyses of animal studies can significantly improve the translational potential of future clinical trials by informing optimal research design (Murphy and Murphy 2010). Through systematic synthesis of existing evidence, such analyses yield comprehensive insights into acupuncture's therapeutic effects on diabetes‐induced cognitive dysfunction in animal models, thereby generating evidence‐based recommendations for both subsequent preclinical studies and clinical trial development.

## 2 Materials and Methods

This study is reported based on the Preferred Reporting Items for Systematic Reviews and Meta‐Analyses (PRISMA) guidelines. In addition, this study is registered in the International Prospective Register of Systematic Reviews (PROSPERO) trial registry (CRD42025638940).

### 2.1 Search Strategy

From database establishment to January 1, 2025, seven databases were searched including PubMed, Web of Science, Embase, Ovid Technologies (OVID), SinoMed, China National Knowledge Infrastructure (CNKI), and Wanfang. The search scope is limited to literature published in both Chinese and English. Search terms were restricted using MESH terms for diseases, interventions, and animal type (“diabetic cognitive impairment” OR “diabetic cognitive dysfunction” OR “diabetic cognitive decline”“Acupuncture” OR “Electroacupuncture” “acupuncture”“db/db” “Rat” OR “Mice” OR“Animals”). The search process was completed jointly by two authors, and when there were discrepancies, the third author was consulted to reach a consensus.

### 2.2 Inclusion and Exclusion Criteria

Initially, the studies that can be included are determined, and the inclusion criteria are as follows: (1) Animal models: studies utilizing validated DCI models. (2) Interventions: the study group adopted acupuncture treatment, including but not limited to traditional acupuncture therapy, electroacupuncture, and other widely used acupuncture therapies. (3) Outcome indicators: the main outcome indicators include fasting blood glucose (FBG) and behavioral tests. Exclusion criteria are as follows: (1) unavailable full‐text publications, (2) duplicate studies or datasets, (3) in vitro or cell culture studies, and (4) animals not subjected to cognitive behavioral tests.

### 2.3 Data Extraction

All literature retrieved from the database was imported into EndNote 21, and duplicates were removed. Two researchers then independently performed data extraction and quality assessment after reviewing the abstracts. The extracted data included the title, first author, and publication year; animal species, number, and gender in the treatment and control groups; modeling methods; interventions, including acupuncture treatment time and method; and outcome indicators. If data were presented in figures or tables, the corresponding authors were contacted to obtain the original data. In cases of nonresponse, Web PlotDigitizer software was used for data estimation. The extracted information was subsequently organized into tables and figures.

### 2.4 Literature Quality Assessment

We assessed the quality of the included studies using the SYRCLE Risk of Bias (RoB) tool, which is specifically designed for animal studies (Hooijmans et al. 2014). The evaluation included the following domains: (1) sequence generation, (2) baseline characteristics, (3) allocation concealment, (4) random housing, (5) blinding of caregivers and investigators, (6) blinding of outcome assessment, (7) incomplete outcome data, (8) selective outcome reporting, and (9) other sources of bias. The results of the quality assessment were visualized using Review Manager 5.3.

### 2.5 Data Analysis

Data analysis and forest plot generation were conducted using Stata 14.0. Continuous variables were analyzed using standardized mean differences (SMDs), and heterogeneity was assessed using the *I* ^2^ statistic. When *I* ^2^ exceeded 50%, a random effects model was applied; otherwise, a fixed effects model was used. All outcomes included 95% confidence intervals (CIs). Subgroup analyses were conducted to identify potential sources of heterogeneity, and sensitivity analyses along with publication bias assessments were also performed using Stata 14.0. A *p*‐value < 0.05 was considered statistically significant.

## 3 Results

### 3.1 Literature Search Results

**FIGURE 1:**
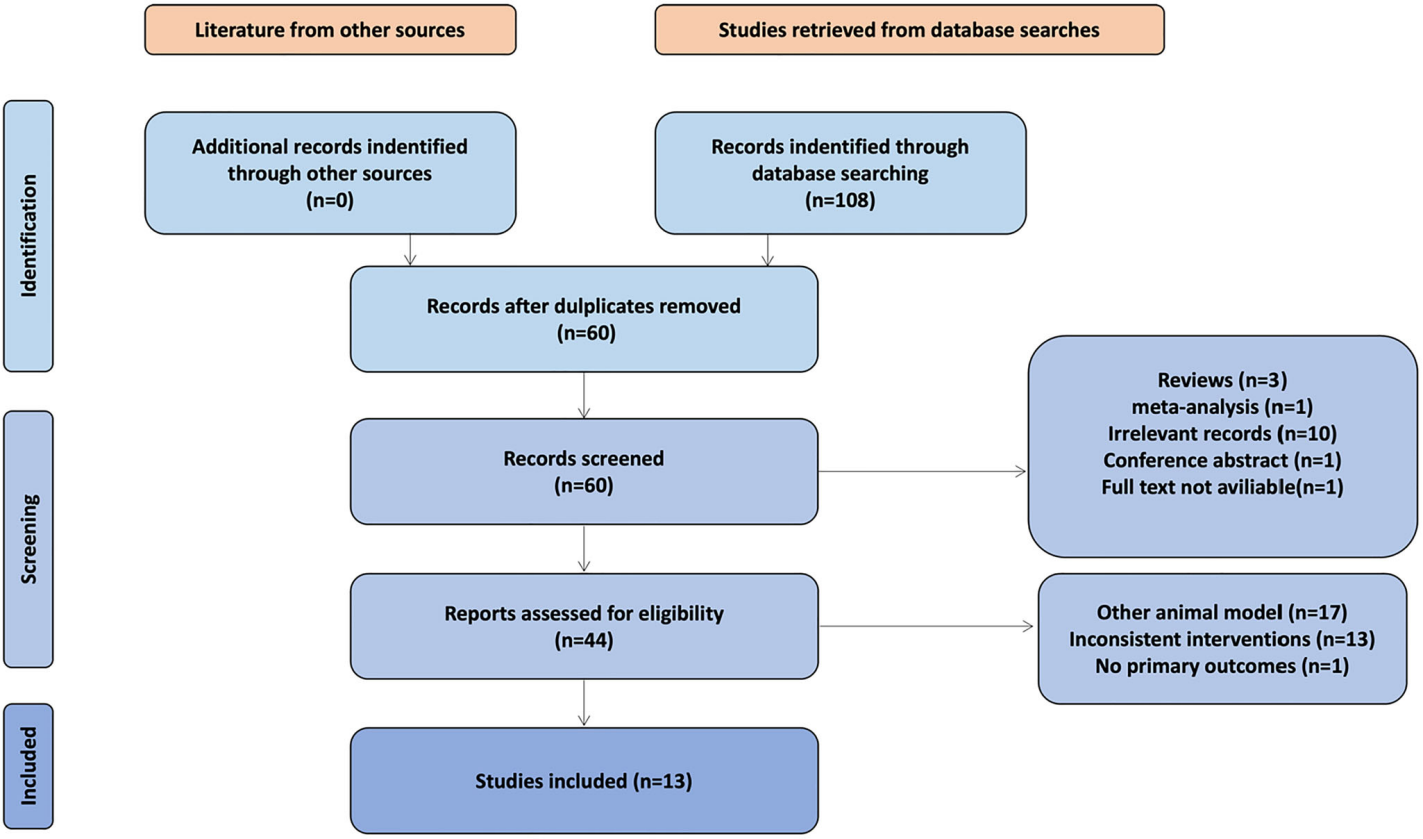
Process of literature screening.

A total of 108 articles were initially retrieved using a predefined search strategy, and 48 duplicates were subsequently removed. After independent screening of titles and abstracts by two reviewers, one meta‐analysis, three reviews, one conference abstract, 10 irrelevant articles, and one article without accessible full text were excluded. Full‐text screening led to the exclusion of 17 studies that did not use DCI animal models, 13 with unmatched interventions, and one lacking primary outcome indicators. Ultimately, 13 studies met the inclusion criteria and were included in the final analysis (Figure 1).

### 3.2 Characteristics of Included Literature

In the subsequent analysis, 13 articles were included, encompassing a variety of study subjects, interventions, and outcome indicators. Specifically, these studies examined different metabolic and cognitive markers, utilized various acupuncture techniques and treatment durations, and employed diverse animal models. A total of 294 animals were analyzed, with 148 in the treatment group and 146 in the control group. The included studies comprised four published in English (Cao et al. 2020; Ge et al. 2022; M. Li et al. 2023; Soligo et al. 2017) and nine in Chinese (Deng et al. 2024; He et al. 2022; Ma et al. 2009, 2008; Pan et al. 2018; L. Wang 2023; W. Wang and Wang 2022; Xu 2002; Aea 2020). The earliest publication date among the 13 studies was 2008, and the most recent was 2024.

Of the 13 included studies, 11 utilized rat models—nine with Sprague–Dawley (SD) rats (Cao et al. 2020; Ge et al. 2022; Soligo et al. 2017; Deng et al. 2024; He et al. 2022; Pan et al. 2018; L. Wang 2023; Xu 2002; Aea 2020) and two with Wistar rats (Ma et al. 2009, 2008). The remaining two studies (M. Li et al. 2023; W. Wang and Wang 2022) used db/db mice. All studies, except one (Soligo et al. 2017), reported the initial weight range of the animals. For rats, weights ranged from 150 to 300 g; for mice, 38 ± 2 g to 40 ± 4 g. Except for the two studies using db/db mice, all others reported the modeling methods. One study (Xu 2002) used alloxan monohydrate, while the remaining 10 employed streptozotocin (STZ) injections. Among those, six studies (Cao et al. 2020; Ge et al. 2022; Deng et al. 2024; He et al. 2022; L. Wang 2023; Aea 2020) used a high‐fat, high‐sugar diet prior to STZ injection, and four (Soligo et al. 2017; Ma et al. 2009, 2008; Pan et al. 2018) used STZ alone. All studies involving STZ‐induced diabetes models described the injection dosage in detail. STZ doses ranged from 25 to 200 mg/kg, with five studies (Cao et al. 2020; Ge et al. 2022; Deng et al. 2024; He et al. 2022; Aea 2020) using 25 mg/kg. One study (Xu 2002) used FBG ≥ 11 mmol/L as the modeling criterion; others used random blood glucose. Among them, one study (Soligo et al. 2017) required > 300 mg/dL, another (Pan et al. 2018) > 350 mg/dL (Type 1 diabetes), and the rest ≥ 16.7 mmol/L.

Regarding interventions, 10 studies (Cao et al. 2020; Ge et al. 2022; M. Li et al. 2023; Soligo et al. 2017; Deng et al. 2024; He et al. 2022; Ma et al. 2009; L. Wang 2023; W. Wang and Wang 2022; Xu 2002) mentioned using a fixed method without other treatment for the control group, while the other three studies (Ge et al. 2022; Ma et al. 2008; Pan et al. 2018) did not mention the treatment measures for the control group. All studies mentioned the acupoint names used in acupuncture. Eight studies (Cao et al. 2020; Ge et al. 2022; M. Li et al. 2023; Soligo et al. 2017; Deng et al. 2024; Pan et al. 2018; L. Wang 2023; Aea 2020) used the Zusanli (ST36) acupoint, and nine studies (Ge et al. 2022; M. Li et al. 2023; Deng et al. 2024; Ma et al. 2009, 2008; Pan et al. 2018; L. Wang 2023; W. Wang and Wang 2022; Xu 2002) used the Baihui (GV20) acupoint. Among the 13 studies, the study with the fewest acupoints (Ma et al. 2009, 2008) used two acupoints (Baihui, Dazhui), and the studies with the most acupoints used were M. Li et al. (2023) and W. Wang and Wang (2022), both using nine acupoints. Detailed information about the characteristics is summarized in Table 1.

**TABLE 1:** Characteristics of included studies.

Study (year)	Species (sex, weight, age); *n* = treatment/control	Modeling method and standard	Acupuncture intervention (waveform, electricity, frequency, time of duration); course	Outcome
Cao et al. (2020)	Sprague–Dawley rats (male, 160 ± 20 g, 100 days); 10/10	Intraperitoneal injection of STZ (25 mg/kg)+ HSFD BG ≥ 16.7 mmol/L	EA (continuous wave, 1 mA, 10 Hz, 20 min); 4 weeks	FBG MWM Bax Caspase‐3 Bcl‐2
Deng et al. (2024)	Sprague–Dawley rats (male, 200–250 g); 12/12	Intraperitoneal injection of STZ (25 mg/kg)+ HSFD BG ≥ 16.7 mmol/L	EA (dilatational wave, 1 mA, 2 Hz/10 Hz, 20 min); 4 weeks	FBG MWM PI3K p‐PI3K Akt p‐Akt CERB p‐CERB Caspase‐3 Bax Bcl‐2
Ge et al. (2022)	Sprague–Dawley rats (male, 200 ± 20 g, 3 months); 8/8	Intraperitoneal injection of STZ (25 mg/kg)+ HSFD BG ≥ 16.7 mmol/L	EA (continuous wave, 1 mA, 15 Hz, 30 min); 4 weeks	FBG MWM Aβ1‐42 Lamp2 COXIV Beclin‐1 Bcl‐2 LC3 P62 Disc1
He et al. (2022)	Sprague–Dawley rats (male, 160 ± 20 g); 9/9	Intraperitoneal injection of STZ (25 mg/kg)+ HSFD BG ≥ 16.7 mmol/L	EA (continuous wave, 1 mA, 10 Hz, 20 min); 4 weeks	FBG MWM Tau NF‐_K_B p65 Aβ1‐42
M. Li et al. (2023)	db/db mice (male, 40 ± 4 g, 8 weeks); 15/15		EA (dilatational wave, 2 Hz/10 Hz, 20 min); 4 weeks	FBG MWM INS TC TG Bax Bcl‐2 Capase‐3 GRP78 IRE1a TRAF2 ASK1 IRS1 P‐IRS1 PI3K P‐PI3K AKT p‐AKT GSK3b p‐GSK3b Aβ1‐42 p‐Tau
Soligo et al. (2017)	Sprague–Dawley rats (female, 50 days); 7/7	Intraperitoneal injection of STZ (65 mg/kg) BG ≥ 300 mg/dL	EA (0.8–1.0 mA, 2 Hz frequency with 0.1‐s, 80‐Hz burst pulses)	FBG MWM ProNGF M1AChR M2AChR LTP
Ma et al. (2008)	Wistar rats (male, 250–300 g); 10/9	Intraperitoneal injection of STZ (60 mg/kg) BG ≥ 16.7 mmol/L	EA (dilatational wave, 30 A, 15 min); 4 weeks.	FBG MWM NT‐3 NGF BDNF
Ma et al. (2009)	Wistar rats (male, 250–300 g); 10/9	Intraperitoneal injection of STZ (60 mg/kg) BG ≥ 16.7 mmol/L	EA (dilatational wave, 30 A, 15 min); 4 weeks	1. FBG 2. MWM 3. CTGF
Pan et al. (2018)	Sprague–Dawley rats (male, 222.4–263.1 g, 8 weeks); 18/18	Intraperitoneal injection of STZ (200 mg/kg) BG ≥ 350 mg/dL	EA (2–4 V, 2 Hz, 20 min); 30 times	FBG INS MWM
L. Wang (2023)	Sprague–Dawley rats (male, 150 ± 20 g, 3 months); 10/10	Intraperitoneal injection of STZ (30 mg/kg)+ HSFD RBG ≥ 16.7 mmol/L	EA (continuous wave, 1 mA, 15 Hz, 15 min); 4 weeks	FBG MWM Beclin1 P62 LC3‐II/LC3‐I
W. Wang and Wang (2022)	db/db mice (38 ± 2 g, 8weeks); 15/15		EA (dilatational wave, 1 mA, 2 Hz/10 Hz, 20 min); 4 weeks	FBG MWM IREα JNK Bcl‐2
Xu (2002)	Sprague–Dawley rats (male, female, 180–220 g); 14/14	Intraperitoneal injection of alloxan FBG ≥ 11 mmol/L	EA (dilatational wave, 30 mA, 15 min); 6 weeks	FBG MWM Glu Asp Gly GABA
Yuan et al. (2020)	Sprague–Dawley rats (male, 60 ± 20 g,); 10/10	Intraperitoneal injection of STZ (25 mg/kg)+ HSFD BG ≥ 16.7 mmol/L	EA (continuous wave, 1 mA, 10 Hz, 20 min); 4 weeks	FBG MWM IL‐6 IL‐1β TNF‐α p38 MAPK STAT3 p‐p38 MAPK p‐STAT 3

### 3.3 Quality Assessment of Included Literature

**FIGURE 2:**
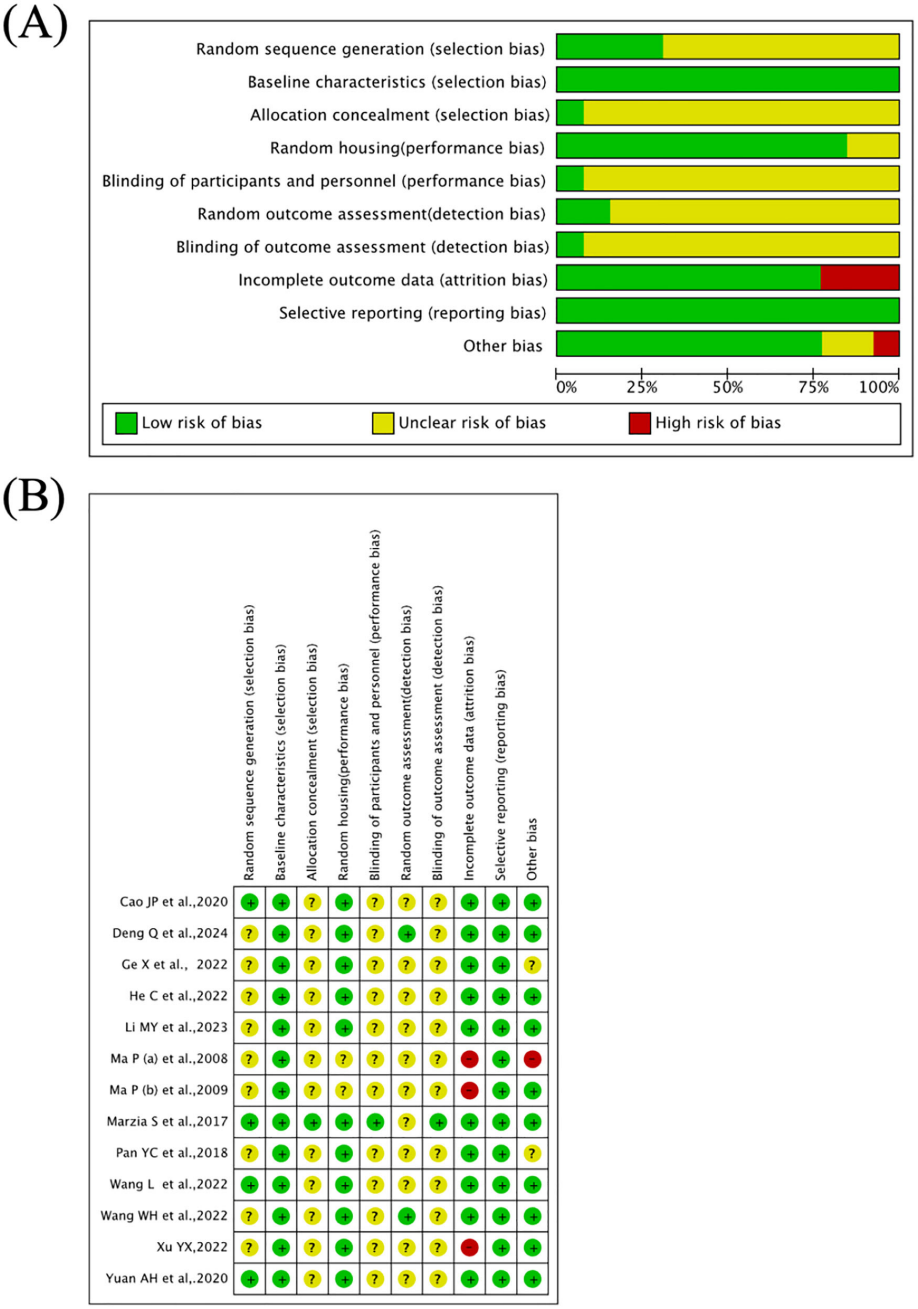
Quality assessment. (A) Risk of bias graph. (B) Risk of bias summary.

We conducted a quality assessment of all included studies. All studies mentioned randomization, and only four studies (Cao et al. 2020; Soligo et al. 2017; L. Wang 2023; Aea 2020) provided specific details on their randomization methods. Baseline characteristics were reported in all studies. Only one study (Soligo et al. 2017) met the criteria for allocation concealment, blinding of participants/personnel (intervention), and blinding of outcome assessment. Two studies (Ma et al. 2009, 2008) did not describe the housing conditions. Two studies (Deng et al. 2024; W. Wang and Wang 2022) reported random selection of animals for experimentation. Three studies (Ma et al. 2009, 2008; Xu 2002) reported missing data, and no score was obtained for data completeness. All articles did not selectively report outcomes. Among the factors that could lead to bias, three studies (Ge et al. 2022; Ma et al. 2008; Pan et al. 2018) did not give treatment to the control group, such as fixing them on the mouse board without taking measures like the intervention group, which could lead to biased results. The quality of literature varied, with scores ranging from 2 to 9 points. Seven studies (Ge et al. 2022; M. Li et al. 2023; He et al. 2022; Ma et al. 2009, 2008; Pan et al. 2018; Xu 2002) scored no more than 5 points. The quality of studies on acupuncture treatment for DCI needs improvement. The quality assessment of literature is shown in Figure 2A,B.

### 3.4 Meta‐Analysis Results

#### 3.4.1 Fasting Blood Glucose

**FIGURE 3:**
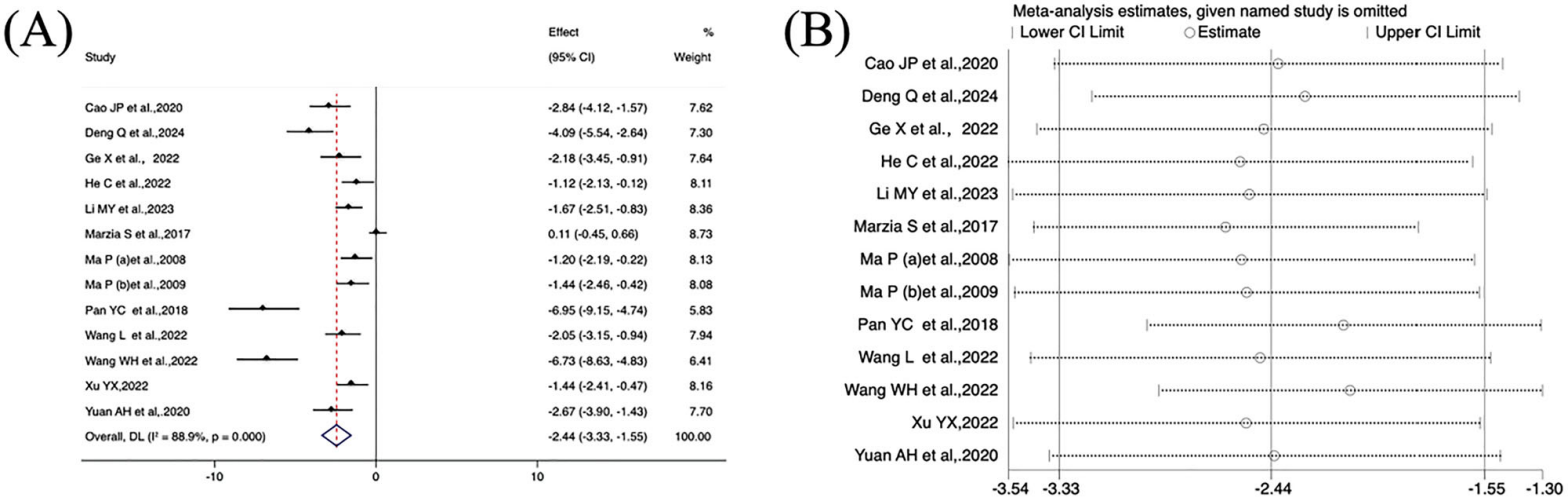
(A) Forest plot of FBG. (B) Sensitivity analysis. CI, confidence interval.

All 13 studies measured FBG, with a total of 294 animals. Acupuncture significantly reduced blood glucose levels in animals [SMD = −2.44, 95% CI (−3.33, −1.55); *I* ^2 ^= 88.9%] (*p *= 0.000) (Figure 3A). The results of the studies showed significant heterogeneity. Due to significant heterogeneity, we chose a random effects model for analysis. Sensitivity analysis was also conducted to explore the source of heterogeneity, but the source was not determined (Figure 3B). Further subgroup analysis was performed using animal models, electroacupuncture treatment time, and the number of acupoints per treatment session, and the results are shown in Table 2. When the electroacupuncture treatment time was less than 15 min, the heterogeneity was 0, indicating that a fixed effects model could be used. In contrast, when the electroacupuncture treatment time was greater than 15 min, there was higher heterogeneity (*I* ^2^ = 85%). These findings imply that treatment duration may modulate the hypoglycemic effect of electroacupuncture in DCI mice. However, the effect of animal models and the number of acupoints per treatment session on heterogeneity was not significant.

**TABLE 2:** Subgroup analysis of FBG.

Subgroup	SMD (95% CI)	*I* ^2^ (%)
Animal		
Mouse	−4.01 [−8.83, −0.81], *p* = 0.10	95
Rat	−2.07 [−2.94, −1.20], *p *< 0.00001	86
Electroacupuncture treatment duration		
Less than or equal to 15min	−1.44 [−1.95, −0.93], *p *< 0.00001	0
More than 15 min	−3.20 [−4.38, −2.01], *p *< 0.00001	85
Number of acupoints in electroacupuncture treatment		
More than 3 points	−3.06 [−4.49, −1.63], *p *< 0.001	85
Less than or equal to 3 points	−1.87 [−2.88, −0.85], *p* = 0.003	87

#### 3.4.2 Morris Water Maze

The Morris water maze (MWM) test is a widely used behavioral assay for evaluating learning and memory functions in animal models. The results of the water maze test in this study include escape latency time and platform crossing frequency. All 13 included studies assessed escape latency, involving a total of 287 animals. Six studies (Cao et al. 2020; Ge et al. 2022; M. Li et al. 2023; Deng et al. 2024; L. Wang 2023; W. Wang and Wang 2022) conducted platform crossing frequency tests. Additionally, two studies (Ge et al. 2022; M. Li et al. 2023) measured the percentage of swimming distance within the target quadrant, and one study (Soligo et al. 2017) assessed the total swimming distance in the target quadrant.

##### 3.4.2.1 Escape Latency Time

**FIGURE 4:**
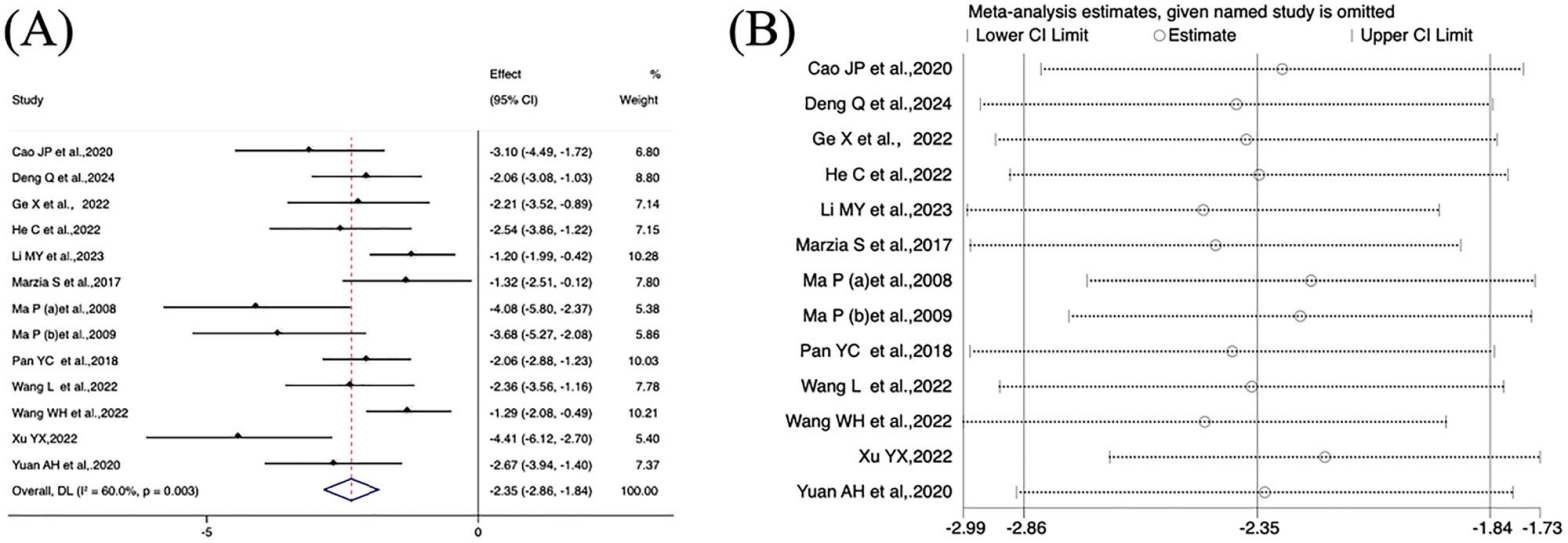
(A) Forest plot of escape latency. (B) Sensitivity analysis.

In terms of escape latency time, the meta‐analysis revealed substantial heterogeneity (*I* ^2^ = 60%, *p* = 0.003). A random effects model was applied to calculate the effect size, yielding a significant result [SMD = −2.35, 95% CI (−2.86, −1.84)], *p *< 0.001 (Figure 4A). Sensitivity analysis did not reveal the source of heterogeneity (Figure 4B), but according to the subgroup analysis results, animal models, electroacupuncture treatment duration, and acupoint quantity were all sources of heterogeneity (Table 3). Overall, the findings suggest that electroacupuncture significantly enhances learning and memory performance in animal models.

**TABLE 3:** Subgroup analysis of escape latency.

Subgroup	SMD (95% CI)	*I* ^2^ (%)
Animal		
Mouse	−1.24 [−1.80, 0.68], *p *< 0.0001	40
Rat	−2.59 [−3.09, 2.10], *p *< 0.00001	0
Electroacupuncture treatment duration		
Less than or equal to 15min	−3.38 [−4.13, 2.63], *p *< 0.00001	39
More than 15 min	−1.89 [−2.25, 1.54], *p *< 0.00001	36
Number of acupoints in electroacupuncture treatment		
More than 3 points	−1.63 [−2.06, −1.20], *p *< 0.00001	15
Less than or equal to 3 points	−2.82 [−3.52, −2.13], *p *< 0.00001	55

##### 3.4.2.2 Platform Crossing Frequency

**FIGURE 5:**
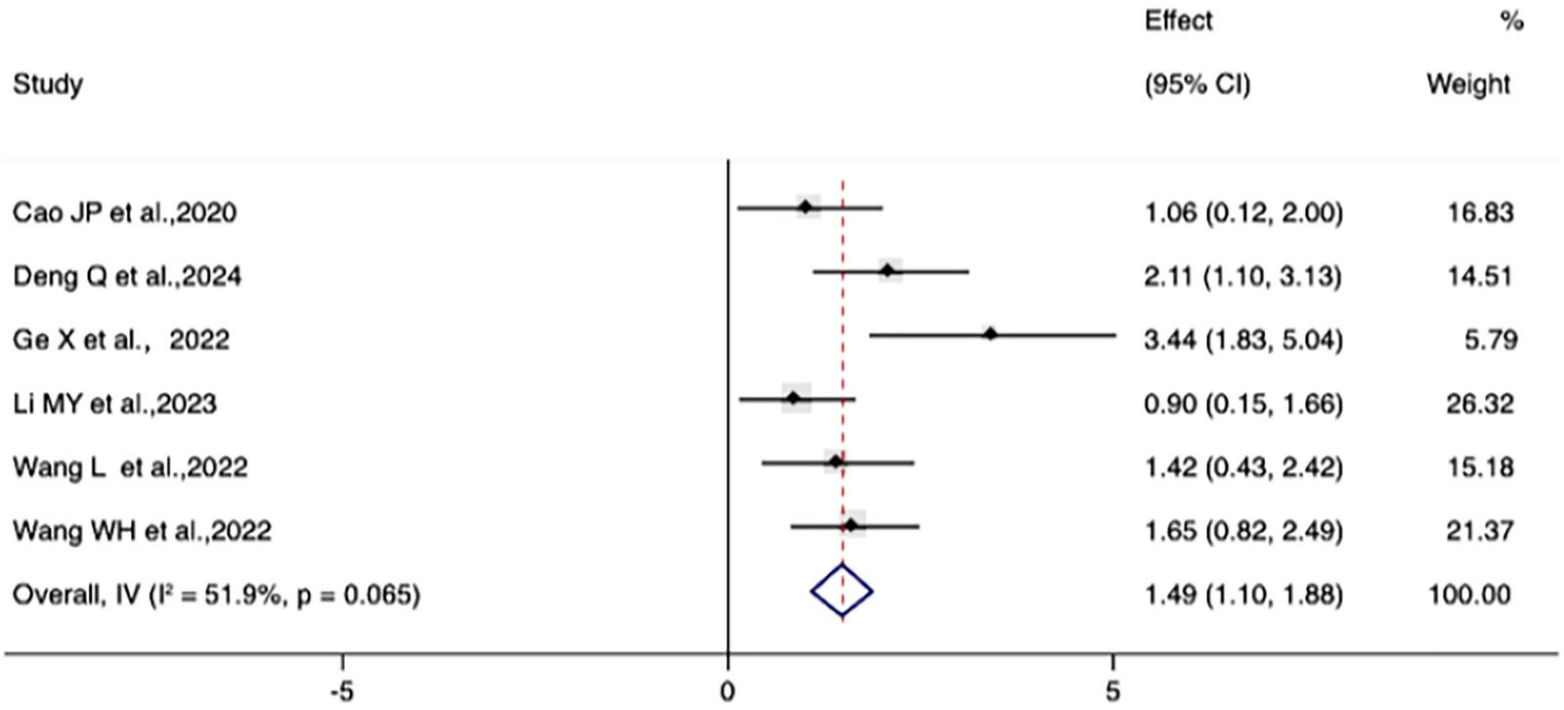
Forest plot of platform crossing frequency.

For platform crossing frequency, the meta‐analysis showed moderate heterogeneity (*I* ^2^ = 51.9%, *p* = 0.065), and a random effects model was used. The results indicated a significant effect of electroacupuncture [SMD = 1.49, 95% CI (1.10, 1.88)], *p *< 0.001(Figure 5), indicating that electroacupuncture treatment can increase the number of platform crossings by animals, and spatial memory ability is improved.

#### 3.4.3 Weight

Five studies (Ge et al. 2022; M. Li et al. 2023; Soligo et al. 2017; W. Wang and Wang 2022; Xu 2002) examined the effects of electroacupuncture on body weight; however, these studies demonstrated extremely high heterogeneity (*I* ^2^ = 93.4%). Subgroup analysis failed to identify significant moderating variables (*I* ^2^ > 90%), and thus a pooled effect size was not reported. Sensitivity analysis showed that even after excluding two outlier studies (Ge et al. 2022; Xu 2002), substantial heterogeneity remained (*I* ^2^ = 66.3%). These findings suggest that the impact of electroacupuncture on body weight may be influenced by complex factors, such as the type of diabetes model and the duration of the intervention.

#### 3.4.4 Funnel Plot Analysis

**FIGURE 6:**
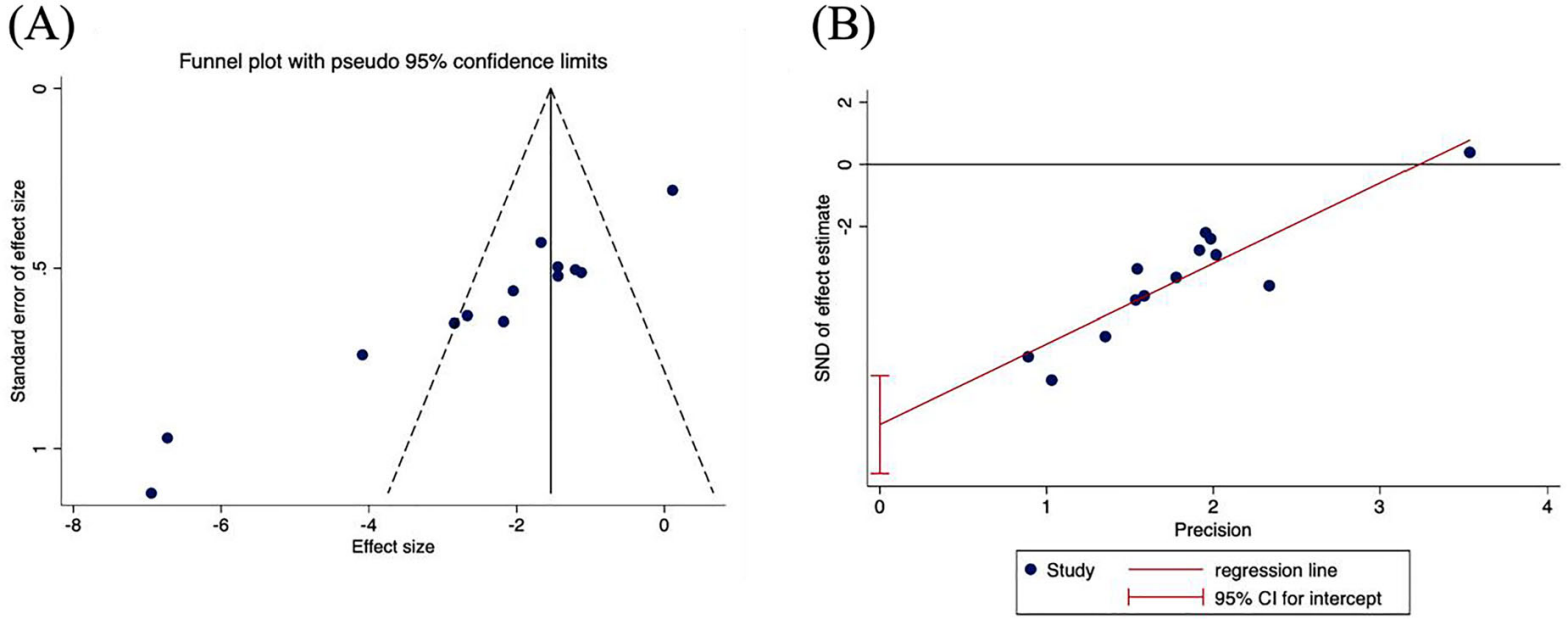
(A) Funnel plot. (B) Egger's bias test graph.

A funnel plot analysis was conducted to evaluate publication bias across the 13 included studies. The asymmetry observed in the funnel plot suggests a potential presence of publication bias (Figure 6A). To further assess this, Egger's test was performed, yielding a *p*‐value of 0.000. Since a *p*‐value < 0.05 indicates significant publication bias (Figure 6B), these results collectively suggest that the included studies may indeed be subject to publication bias.

#### 3.4.5 Tau Protein

Two studies included in the research discussed the effects of acupuncture on tau protein. He et al. (2022) observed that the expression and mRNA content of tau protein in the electroacupuncture group were lower than those in the model group. M. Li et al. (2023) suggested that the phosphorylation level of tau protein was increased in the model group, while electroacupuncture could improve the high phosphorylation of tau protein.

## 4 Discussion

Systematic reviews and meta‐analyses have become indispensable evidence in preclinical and clinical research (Hernandez et al. 2020). Meta‐analysis of animal models provides a more objective and rigorous assessment of the effectiveness of interventions, surpassing the limitations of traditional narrative reviews related to animal studies and reducing unnecessary animal experiments in preclinical research (Sandercock and Roberts 2002; Hooijmans et al. 2012).

This study demonstrates that acupuncture therapy exhibits beneficial effects in lowering blood glucose levels and improving learning and memory functions in animal models. Despite significant heterogeneity, subgroup and sensitivity analyses revealed that the choice of animal model, duration of acupuncture treatment, and number of acupoints are key factors influencing therapeutic efficacy. In the escape latency test, rats exhibited superior performance [SMD = −2.59, 95% CI (−3.09, −2.10)], consistent with previous findings (Fan and Zhang 2024), which may stem from functional differences in hippocampal structure between rats and mice (McNamara et al. 1996). Notably, subgroup analysis showed that when the acupuncture duration was < 15 min and the number of acupoints was ≤ 3, the improvement in cognitive function was more pronounced. This phenomenon contrasts sharply with the cumulative dose effect observed in acupuncture's hypoglycemic action, suggesting a potential differential “dose‐response” relationship between neurological and metabolic systems.

From a neurobiological perspective, short‐term, low‐frequency stimulation may exert optimal effects through the following mechanisms: (1) moderate activation of microglia, avoiding excessive stimulation‐induced inflammatory responses (Hwang et al. 2025); (2) pulsatile release of brain‐derived neurotrophic factor (BDNF) in the hippocampal region enhances synaptic plasticity, whereas prolonged stimulation may lead to receptor desensitization (Park and Poo 2013); and (3) alignment with the “spike‐timing‐dependent plasticity (STDP)” theory, where brief and precise stimulation more readily induces long‐term potentiation (LTP). Traditional Chinese medicine (TCM) theory may explain this as *“shǎo huǒ heng qì, zhuàng huǒ shí qì”* (*Suwen·Yinyang Yingxiang Dalun*), where moderate stimulation activates meridian *qi*, while excessive stimulation depletes vital energy. This finding carries important clinical implications: for cognitive dysfunction, acupuncture may exhibit a “minimum effective dose,” contrasting with the cumulative dose effect observed in metabolic disorders (e.g., diabetes). Future research should focus on the following: (1) defining stimulation intensity thresholds for different neural targets and (2) investigating synergistic/antagonistic effects of acupoint combinations.

In the selection of acupoints, the most frequently used acupoint is Baihui (GV20), followed by Zusanli (ST36). Baihui, as a head acupoint, can regulate brain nerve function and enhance cognitive abilities. This may be achieved by activating the cholinergic system, upregulating the expression of brain‐derived neurotrophic factor (BDNF) and cAMP‐response element‐binding protein (CREB), and protecting neurons (Lee et al. 2014). For Zusanli (ST36), many studies have found that combining it with Zusanli can enhance cognitive function (F. Guo et al. 2023; Z. G. Wang et al. 2023), which may be related to its anti‐inflammatory effects (Torres‐Rosas et al. 2014). In a recent study related to artificial intelligence, it was revealed that acupuncture or sham acupuncture at Zusanli (ST36) can alter electroencephalograms representing brain activity (Zhang et al. 2024), further confirming the relationship between Zusanli and the brain.

Although the number of clinical studies on acupuncture treatment for DCI is limited, existing evidence suggests that acupuncture can increase hippocampal volume, improve neuronal structure, and reduce brain oxidative stress levels by enhancing superoxide dismutase (SOD) activity, decreasing peripheral blood malondialdehyde (MDA) content, and upregulating BDNF expression (Zhu et al. 2021). Further supporting this, Yao et al. (2023) demonstrated that the “Adjust Zang‐fu and Arouse Spirit” electroacupuncture method significantly improves cognitive function in diabetic patients by modulating levels of *N*‐acetylaspartate (NAA) in the left basal ganglia, myo‐inositol (MI) in the right basal ganglia, and γ‐aminobutyric acid (GABA) in the right basal ganglia. In terms of acupoint selection, the “Adjust Zang‐fu and Arouse Spirit” electroacupuncture method commonly employs a combination of Baihui (GV20), Shenting (GV24), Zusanli (ST36), Sanyinjiao (SP6), Hegu (LI4), Taichong (LR3), and back‐shu points (Feishu BL13, Pishu BL20, and Shenshu BL23). These acupoints collectively improve cognitive function by regulating nervous system activity, enhancing Qi and blood circulation, and strengthening the functions of the Zang‐fu organs. For instance, Baihui and Shenting enhance learning and memory by modulating the frontal‐hippocampal neural circuit, while Zusanli and Sanyinjiao exert synergistic effects through anti‐inflammatory and metabolic regulation. Additionally, the “Four Gates” acupoint combination (Hegu and Taichong) further enhances therapeutic efficacy by harmonizing Qi and blood and activating brain regions associated with cognition. Despite the promising results of current clinical studies demonstrating the significant efficacy of acupuncture in diabetic patients, the limited number of studies and insufficient sample sizes constrain the reliability of these findings.

Current preclinical animal studies provide important insights for clinical acupuncture practice, particularly regarding acupoint selection and parameter settings. Based on existing evidence, we propose an optimized protocol for clinical research: For core acupoint combinations, Baihui (GV20) and Zusanli (ST36) serve as primary points, complemented by Shenting (GV24) and Sanyinjiao (SP6) as secondary points. This combination retains high‐frequency effective acupoints while incorporating multi‐target synergistic effects for cognitive regulation. Regarding treatment parameters optimization, we recommend a single session duration of approximately 15 min (animal studies indicate this duration yields optimal neuroprotective effects), with a treatment frequency of once daily in the acute phase and every other day in the recovery phase while maintaining appropriate stimulation intensity based on patient tolerance and sustained Deqi sensation. For treatment course design, a basic regimen of four consecutive weeks (corresponding to the critical time point for neurological improvement in animal studies) is suggested, followed by an 8‐week consolidation phase with twice‐weekly sessions to maintain therapeutic effects. This protocol integrates traditional acupoint theory with modern research findings, ensuring clinical feasibility while maximizing the replication of effective intervention models from animal studies. Subsequent research should employ randomized controlled and blind method designs focusing on objective outcomes such as hippocampal volume and serum BDNF levels.

Acupuncture, as a non‐pharmacological intervention, avoids many adverse reactions caused by drugs, such as indigestion and vomiting (Witt et al. 2009; Zhao et al. 2011). Acupuncture treatment can usually serve as an alternative or adjunct to drug therapy, especially for chronic diseases or conditions requiring long‐term treatment (Huang et al. 2019; Su et al. 2021; Lai et al. 2020). It can serve as a safe and effective complementary treatment, alleviating the burden of drug therapy. In current animal studies, acupuncture has demonstrated significant efficacy in treating DCI, particularly in improving learning and memory functions and regulating glucose metabolism.

## 5 Limitations

Several important limitations should be noted in this meta‐analysis. First, the number of included studies was limited, and significant heterogeneity was observed in methodological quality. The pooled effect sizes should be interpreted with extreme caution, as these results may not reflect consistent treatment effects across studies. Notably, the lack of randomization or blinding in some trials may have led to an overestimation of treatment effects. Second, our publication bias analysis suggests potential selective reporting of positive results, further indicating that the apparent efficacy of acupuncture might be exaggerated. Third, although rigorous correction methods were applied, the use of graph digitization software for indirect data extraction due to unavailable raw datasets may still introduce measurement inaccuracies. Fourth, the identified significant publication bias suggests that our conclusions may primarily reflect positive outcomes and may not fully represent the entire body of relevant research evidence. Additionally, since the search was restricted to articles published in Chinese and English, language restrictions may have introduced further publication bias.

To address these limitations, we strongly recommend that future preclinical studies adhere to standardized guidelines such as SYRCLE's risk‐of‐bias tool or ARRIVE 2.0 to enhance methodological rigor. Additionally, large‐scale, multicenter trials with transparent protocols (e.g., preregistration) and integrated multi‐omics approaches are warranted to validate acupuncture's mechanisms and optimize its clinical translation for DCI.

## 6 Conclusion

Current evidence suggests that acupuncture may have a positive effect on improving cognitive and glycemic levels in animal models of DCI. However, due to high heterogeneity among studies, the reliability of this conclusion is limited, and more homogenous, high‐quality studies are needed for verification.

## Author Contributions

**Xinyi Jiao**: conceptualization, data curation, investigation, formal analysis, writing – original draft, software, methodology. **Yue Hu**: conceptualization, investigation, formal analysis, writing – original draft. **Guoqing Tian**: writing – review and editing, supervision, funding acquisition, project administration. All authors reviewed the manuscript. All authors read and approved the final version of the manuscript.

## Ethics Statement

The authors have nothing to report.

## Conflicts of Interest

The authors declare no conflicts of interest.

## Peer Review

The peer review history for this article is available at https://publons.com/publon/10.1002/brb3.70783

## Data Availability

The data that support the findings of this study are available from the corresponding author upon reasonable request
